# Advanced Quantification Pipeline Reveals New Spatial and Temporal Tumor Characteristics in Preclinical Multiple Myeloma

**DOI:** 10.21203/rs.3.rs-6596974/v1

**Published:** 2025-05-14

**Authors:** Zhixin Sun, Jacqueline Godbe, Alexander Zheleznyak, Brad Manion, Junhao Hu, Julie Prior, Kathleen Duncan, Ulugbek S. Kamilov, Monica Shokeen

**Affiliations:** 1 Department of Electrical and Systems Engineering, Washington University in St. Louis, Saint Louis, Missouri, USA; 2 Edward Mallinckrodt Institute of Radiology, Washington University School of Medicine, Saint Louis, Missouri, USA

**Keywords:** Multiple Myeloma (MM) Imaging, Bone Segmentation, PET/CT Quantification, Skeletal Lesions

## Abstract

**Background.:**

Radiological imaging plays an indispensable role in both preclinical and clinical studies of multiple myeloma (MM). However, manual quantification in longitudinal small animal PET/CT is limited by annotator bias, signal artifacts from urinary/fecal excretion, and voxel misalignment due to non-rigid registration. To address these challenges and improve characterization of tumor biology, we developed a semi-automated PET/CT quantification pipeline targeting defined regions of interest (ROIs) within the bone marrow-rich mouse skeleton, achieving sub-organ spatial resolution, including in anatomically complex sites such as the pelvis. We applied this MM-specific preclinical pipeline to analyze tumor distribution in a longitudinal molecular PET study using an immunocompetent mouse model of skeletally disseminated MM. An Attention U-Net was trained to segment the thoracolumbar spine, pelvis and pelvic joints, sacrum, and femurs from 2D CT slices. A custom algorithm masked spillover signal from physiological excretion, and a PCA-based projection was used to map tumor distribution along the skeletal axis. Quantification metrics included mean and maximum standardized uptake values (SUV_mean_, SUV_max_) from PET and Hounsfield Units (HU) from CT to assess tumor burden, spatiotemporal tumor distribution, and bone involvement.

**Results.:**

Tumor burden localized preferentially to skeletal regions near joints. Using precise CT-based alignment (DICE = 0.966 ± 0.005), we detected early disease progression and aggressive phenotypes. A marked increase in tumor uptake was observed by day 18 post-implantation, with significant SUV_mean_ increases in the spine (p = 0.012), left/right femurs (p = 0.007/0.006), pelvis and pelvic joints (p = 0.018), and sacrum (p = 0.02). Notably, sex-based differences were identified: female mice showed greater bone loss near the hip joint at later stages, with significant HU_mean_ reductions at days 25 (p = 0.008) and 32 (p = 0.002).

**Conclusions.:**

This pipeline enables reproducible, anatomically precise quantification of region-specific trends in MM progression, including joint-specific lesion tropism and sex-based differences, from longitudinal PET/CT scans. By mitigating common challenges such as excretion artifacts and inconsistent mouse positioning, our approach overcomes limitations of manual analysis and enhances evaluation of tumor biology and treatment response in preclinical models of bone-involved cancers.

## Graphical Abstract.

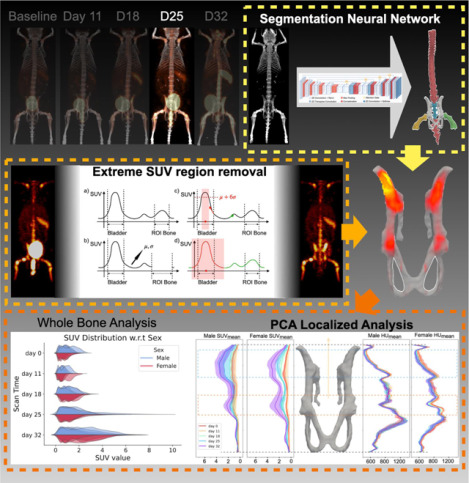

## Introduction.

Cancers involving the bone marrow such as multiple myeloma (MM) are characterized by complex biology, spatial and clonal heterogeneity and frequent relapse. Radiology plays an integral role in MM, preclinically and clinically. Fluorodeoxyglucose (^18^F-FDG) PET/CT is recommended by the International Myeloma Working Group (IMWG) to assess treatment response and detect residual disease in MM, where PET scans help to evaluate lesions, while CT is useful in evaluating lytic bone lesions and fractures [1–3]. More recently, molecularly targeted PET agents such as radiolabeled anti-CD38 antibodies have also been used in preclinical and clinical research settings to evaluate MM progression and treatment responses, while new tracers are under development for potential use in patients [4–7].

Given the complexity of MM as a disease, there is a need for standardized, consistent, and accurate quantification methodologies for evaluating myeloma across time and across mouse models. Temporal (i.e., same subject imaged over time) quantification is limited by three major factors: (1) MM exhibits characteristics of both liquid and solid cancers, presenting as focal, diffuse, or mixed patterns. As such, many lesions do not have clear boundaries, unlike most subcutaneous tumors, which makes repeatable and accurate tumor measurements over PET based boundary difficult. (2) Excretory artifacts from accumulation in urine and feces can affect true tumor signal and frequently change between scans over time. (3) Differences in subject positioning between scans make appropriate registration of features across time, particularly appendages, exceptionally difficult.

Traditional preclinical image analysis of the tumor burden in the skeleton mainly involves manual segmentation of the anatomy of the tissue of interest (e.g. CT based [8], MRI based [9]), followed by the mask mapped to the PET image for further quantification. However, manual image analysis is prone to inter- and intra- annotator bias [10]. The small size of the mouse skeleton and complex anatomy of regions of particular interest in MM (i.e. the vertebral bodies) adds an additional layer of possible error. These challenges can be addressed with the current advancements in segmentation tools. Despite recent advances in automated segmentation [10–19], currently available models are still limited by inability to differentiate between various bones [10, 11, 17], requires different levels of manual interaction [12, 18, 19], or yields suboptimal segmentation results compared to state-of-the-art methods [13, 17]. For these reasons, we trained our own fully automated segmentation model to address the unique pathology of MM.

Once we get the segmentation masks, we can evaluate progressive changes in both standardized uptake value (SUV) and Hounsfield Unit (HU) related metrics in the skeleton, including pelvis, across time points. Due to the pelvis’s proximity to the bladder and rectum, it was necessary to distinguish between artifacts from tracer excreted in urine or feces and true disease progression. While pre-imaging strategies can help mitigate some of the undesirable effects of spillover from excretions [20, 21], post-imaging strategies during analysis are also useful adjuncts and independently valuable for workflow and reducing variables in the experimental design. Therefore, here we propose an automatic post-imaging processing algorithm based on PET.

**Fig. 1:**
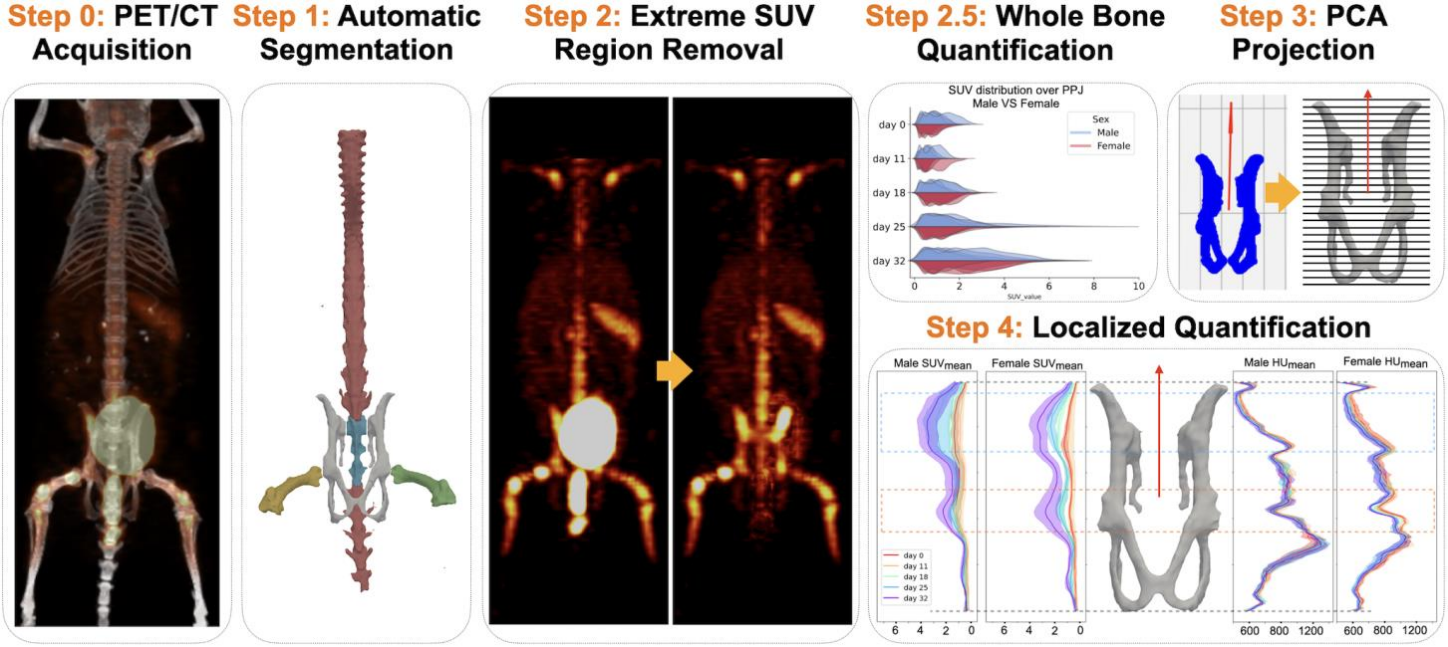
Overview of our pipeline. The region of interest (ROI) is first segmented based on the mouse CT. Then, the extreme SUV region is removed to reduce false positives in affected ROIs. In addition to the regular whole bone analysis, we also propose a PCA-based projection tool to enable localized quantification. We demonstrate the potential of our pipeline for analyzing the development of MM by comparing tumor burden growth among different mice and organs

Longitudinal analysis in MM is useful for evaluating treatment response and serves as a powerful research tool to study the development of myelomatous lesions across different regions. Because of differences in subject positioning between scans, the first step in developing a useful longitudinal analysis tool is to align the region of interest (ROI) across the time points of interest. Recent advancements in non-rigid registration can align the ROI by adjusting the positions of voxels [22, 23]. However, it is difficult to verify whether these positional changes preserve the true relative positions of the signals [24]. To align bones while preserving voxel positions, we assume that the shape of the bones remains constant over time in MM. We then apply principal component analysis (PCA) to find a vector aligned with the direction of the bone. This vector enables us to consistently section the bones into uniform slices, facilitating comparisons across different time points. Metrics such as mean, maximum, percentage of voxels exceeding a threshold, and standard deviation for both PET and CT signals can then be collected and compared longitudinally with appropriate statistical methods. Using this approach, we demonstrate that the areas of high and progressive tracer uptake are observed over the ROIs. The full pipeline we proposed here is described in Fig. 1.

**Fig. 2:**
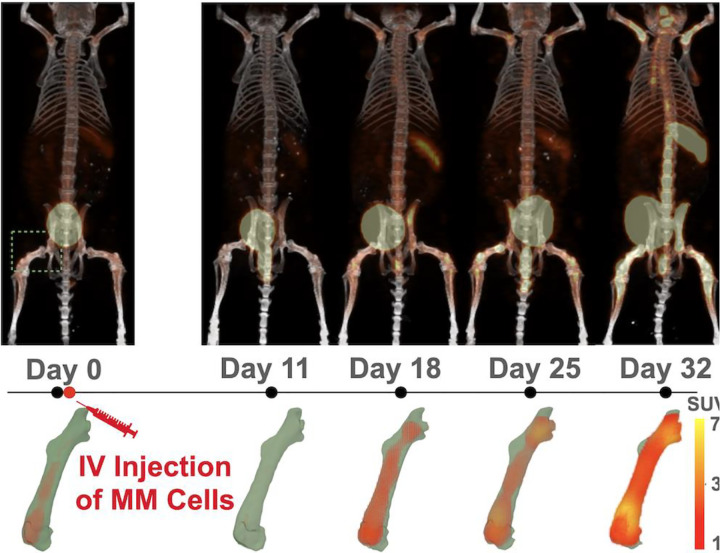
In a longitudinal ^64^Cu-LLP2A PET/CT study, we demonstrated that by accurately segmenting the bone, we can numerically trace how the overlapped SUV values change for each bone ROI over time

In the proof-of-principle study described here, we applied this new tool to evaluate the differences in the progression of MM in immunocompetent male and female mice bearing disseminated tumors (Fig. 2; **Supplementary Fig. S1**).

## Methods.

### Defining the Regions of Interest (ROIs).

**Fig. 3:**
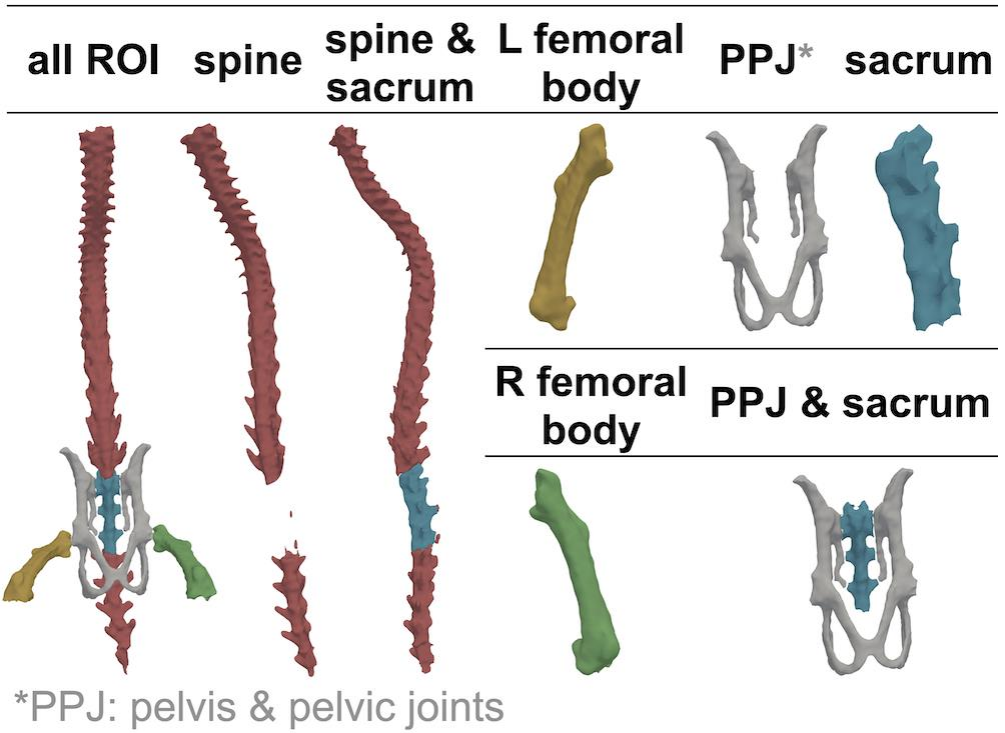
Visual schematic of the 5 key ROIs that were segmented in this study – Spine (red), Pelvis (gray), Sacrum (blue), Left Femur (yellow) and Right Femur (green). The 5 ROIs do not overlap by design; while the sacrum can be combined with spine or pelvis later in the analysis as needed

In this study, we focused on MM lesions that developed in the spine, pelvis & pelvic joints (PPJ), and bilateral femurs (Fig. 3) (**Details in the Supplementary File**).

### Segmentation.

#### Model Structure.

For building our segmentation model, we employed Attention U-Net [25] to segment on each 2D CT slice. As shown in **Supplementary Fig. S2**, Attention U-Net is a variant of U-Net that incorporates learnable attention gates into the skip connections. These gates act as filters, enhancing relevant feature details for improved segmentation while suppressing less informative signals. The output feature of the shallowest attention gate is shown in **Supplementary Fig. S3**.

#### Positional Encoding.

Since we trained our model using 2D sagittal CT views, the slices lacked visual clues for differentiating between the left and right femurs. Therefore, we introduced the same fixed positional encoding as previously used in [26] to encode the relative slice position provided by the 3D mouse CT. This positional encoding vector is concatenated to the final feature layer of the U-Net before an extra convolutional layer, which maps the learned features to the desired classification map.

#### Size of Datasets.

We have 52 labeled 3D CT data for mice scanned under two different energy levels, 60 kvp (n=24) and 80 kvp (n=28), respectively. In Table 1, we report how our training set, validation set, and test set are constructed. More implementation details and ablation study results are provided in the **Supplementary Fig. S1, Supplementary Fig. S2, and Supplementary Table S1**.

### Removal of spillover PET signal from SUV quantification of ROIs.

As we analyzed the statistical metrics for PPJ, we occasionally observed massive fluctuations in values over time. Upon further inspection, we identified these fluctuations as false positives caused by spillover signals from mouse urine and feces. Radiotracer accumulation in these excretions often results in elevated SUV around the bladder and colon, leading to false positives. While these artifacts can be identified based on anatomical location, signal spillover into adjacent structures can hinder accurate assessment of critical pelvic regions.

**Fig. 4:**
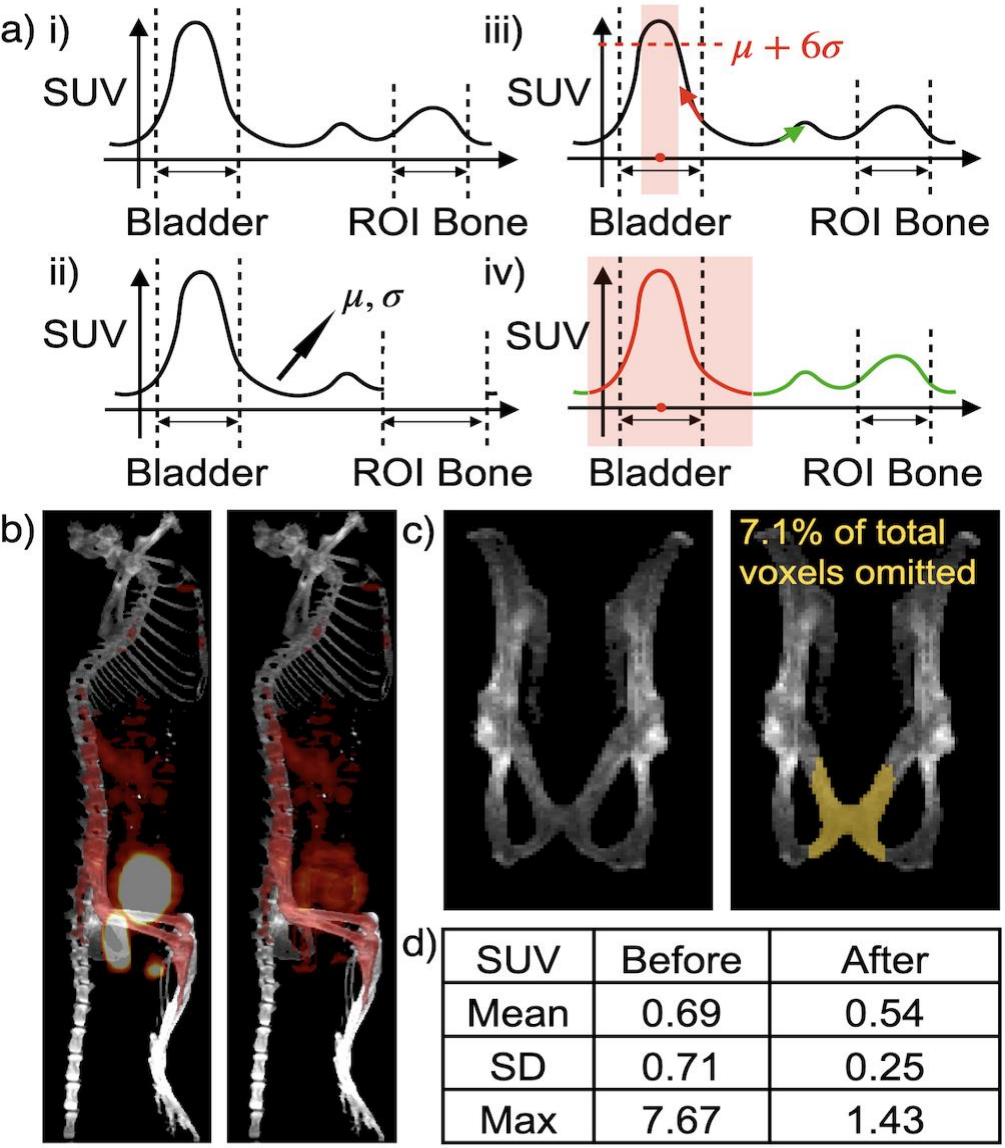
The process and rationale for removing the extreme SUV region. **a)** 1D demonstration of the extreme SUV region removal algorithm: i) the solid curvy line mimics SUV, and the dash bounds the anatomical organ regions. ii) mean and std of non-ROI SUV were computed omitting the padded region of ROI, iii) identification of the extreme SUV regions with a threshold, find the geometric center of that region, and compute SUV gradient for each voxel. iv) continuously remove points where the gradient is pointing away from the extreme SUV region center. **b)** the sagittal projection of fused PET-CT before (left) and after (after) extreme SUV region removal. **c)** the range of PPJ before (left) and after (right) removal, where voxels labeled in yellow (7.1% of the original PPJ) are omitted after removal. **d)** common SUV metrics vary massively before and after extreme SUV region removal over the corresponding range in PPJ

To address this, we propose an algorithm for removing spillover regions with elevated SUV. We demonstrate it in 1D in Fig. 4(a) for illustrative purposes, while the algorithm is inherently designed in 3D. As shown, we first identified regions of abnormally high SUV outside the primary ROIs using a standard deviation-based threshold. Next, we computed the geometric center of each high-SUV region and evaluated the SUV gradient for voxels in the surrounding area. Voxels with gradients oriented toward the region center - rather than away from it - were presumed to be affected by spillover from extreme SUV sources such as the bladder or feces. These voxels were then excluded from downstream analyses to avoid contamination by false-positive signals. Visual result before and after the removal in Fig. 4(b). Fig. 4(c–d) illustrates how the statistical metrics for the same pelvic region can vary with or without the effects of inflated SUV regions. Additional visual results of the extreme region removal from different views are provided in **Supplementary Fig. S4**.

### Localize SUV changes with projection.

In longitudinal studies of MM, we are not only interested in how SUV and HU values change across the entire ROI, but also how these changes correspond to functional or anatomic regions of interest that may provide pathophysiologic insight. Assuming the bone’s shape remains relatively stable over time, we apply PCA to its 3D coordinates to identify the primary anatomical axis. The first principal component, which captures the greatest variance in the data, serves as an estimate of the bone’s longitudinal direction. We can then numerically slice the bone of interest into slices that are perpendicular to the vector, and compute metrics such as mean, std dev and max for each of the slices and plot them along the long axis of the bone. By doing so, we can track signal’s change with respect to their relative location along the bone. To validate this, we first showed in **Supplementary Fig. S5** that, given a mouse’s ROI, the computed vector aligns well visually with the long axis. We note that because the spine is curved and can change shape due to different positioning of the mouse, the long-axis PCA found is not meaningful. Therefore, we did not apply our method to the spine. We then demonstrated that the slicing is consistent across different PET/CT scans for the same mouse by computing the voxel count of each slice **(Supplementary Fig. S6)**. The plot of overlapping slice voxel counts for different scans of the same mouse demonstrates that both the long-axis identification and the subsequent slicing are consistent across different PET/CT scans over time.

### 5TGM1/KaLwRij immunocompetent mouse model.

We used the syngenetic, immunocompetent, disseminated 5TGM1/KaLwRij MM mouse model [27] to evaluate the progression of MM. All mouse experiments were performed in compliance with protocols approved by the Washington University Animal Welfare Committee. All preclinical methods are reported in accordance with ARRIVE guidelines (see **Supplementary File**).

### Radiolabeling, Small Animal PET Imaging and manual PET ROIs.

The mice were imaged longitudinally with ^64^Cu-LLP2A/PET [6] weekly for 5 weeks; details provided in **Supplementary File**. Each mouse served as its own control. That is, mice were imaged prior to tumor injection (week 0) and then once over 4 weeks (once/week) (See Fig. 2).

### Statistical Analysis.

We employed t-tests to investigate MM progression between male and female mice by evaluating changes in SUV and HU metrics from baseline values at different time points. Our proposed projection tool allows us to compare these changes not only across the whole bone but also in selected areas.

## RESULTS.

### Evaluation of the segmentation model.

To evaluate the performance of the segmentation model, we computed the DICE and mIoU score between the 3D predicted mask and the manual labeled mask for 13 mice (80 kvp n=7, 60 kvp n=6) that were put aside during training or validation phase. The average inference time for each mouse is 24.8 s ± 2.6 with a NVIDIA RTX A6000 GPU. Since mice in the longitudinal analysis study were imaged under 80 kvp, we report the performance of the segmentation model on 80 kvp test data in Table 2. In the appendix, we report test performance on 60 kvp mice and present the results of ablation studies comparing different training sets and segmentation models. We showed how a larger training set where we mixed CT taken under different energy levels improved the model’s performance, and how attention gate improved the standard deviation of the test set performance.

### Removal of spillover PET signal from SUV quantification of ROIs.

We evaluated the removal results visually, as shown in Fig. 4(b) and **Supplementary Fig. S4**. Although this removal algorithm requires more rigorous validation in the future, it allows us to inspect the PPJ area more effectively and obtain a more accurate estimation of SUV changes within the PPJ. Fig. 4(c–d) demonstrate that spillover signals from urine and feces can significantly alter statistical metrics, even when they affect a small portion (7.1% of the volume) of the PPJ.

### Longitudinal Data Analysis.

**Fig. 5:**
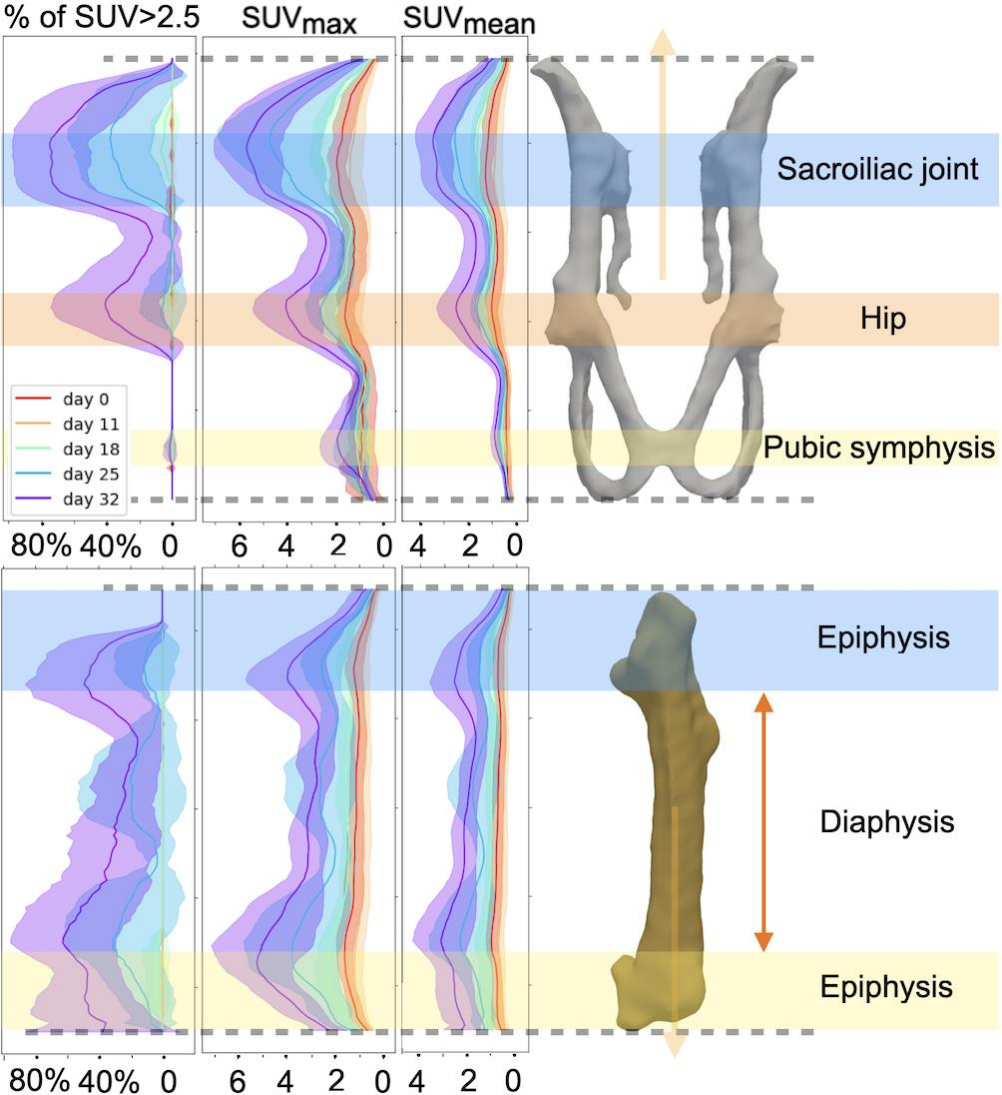
SUV signal changes over time with respect to different locations. Data plots showing: the percentages of voxel that has SUV greater than 2.5 (left), SUV_max_ (middle), and SUV_mean_ (right) change over time with respect to the relative locations in bones for the defined PPJ (top) and the femoral body (bottom). Mean (solid line) and std deviation (shade) of the above metrics of all 8 mice were computed and plotted. Spilled over SUV from mice excretions were masked. As shown, lesions don’t distribute uniformly in bones, with their location preferentially aligned with the location of joints

Fig. 5 shows how SUV_max_, SUV_mean_, and the voxel count of SUV > 2.5 change along the long axis over time for the PPJ and left femur. We observed that the locations where lesions tend to reside correlate with the joint locations. Similar analyses for the right femur, sacrum, and the combination of PPJ and sacrum are provided in **Supplementary Fig. S7**.

**Fig. 6:**
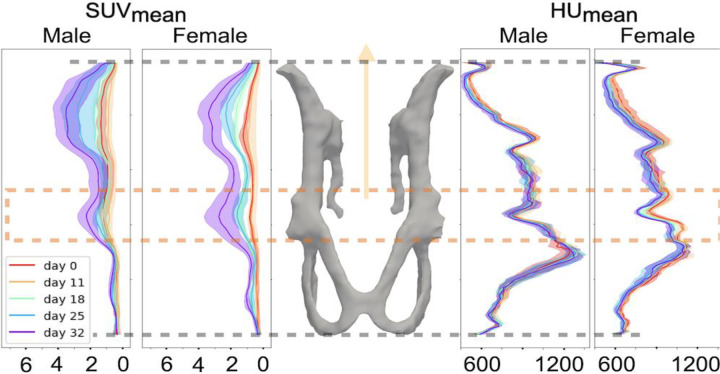
This figure illustrates changes in SUV_mean_ and HU_mean_ for female and male mice at different locations in the PPJ over time. Key observations include: 1) A significant increase in SUV_mean_ for female mice at day 18 (shown in green in the left two plots), while males show little to no change from baseline. 2) Greater bone loss in the acetabular segment of the PPJ for female mice (highlighted by the orange box) compared to males, as shown in the right two plots

Due to differences in pelvic structure and bone density between male and female mice (see **Supplementary Fig. S8**), we also reported how SUV and HU values change locally by sex in Fig. 6. We observed that females tend to experience more bone loss in the acetabular segment of the PPJ (including the acetabulum and femoral head). This is validated using t-test as described later.

### Statistics.

In **Supplementary Fig. S9**, we present our results comparing the distribution of SUV metrics with biological sex as a variable. Statistical analysis was performed after applying the extreme SUV mask for excretory tracer. Initially, we conducted a t-test for the whole bone. The t-test results for changes in SUV metrics from baseline for females and males at day 18 are reported in Table 3. As shown, female mice exhibit more changes from baseline for several SUV metrics within all the traced ROIs. A full set of results for all time points can be found in the **Supplementary Table S2**.

We then conducted a t-test for specific ROIs, as shown in Fig. 6. We manually selected the slices representing the acetabular segment of the PPJ, computed the aggregated metrics over the whole region, and then performed the t-test. The t-test results for changes in PET/CT signals from baseline for females and males are reported in Table 4. These results indicate more aggressive increases in SUV metrics at day 18 and more bone loss at days 25 and 32 in females over the defined area.

### Discussion.

Our segmentation model provides consistent and rapid labeling of regions of interest in seconds, significantly reducing the manual processing time. The extreme SUV region removal algorithm enables us to explore previously inaccessible changes in the pelvis. Consistent segmentation results also allow for the application of PCA to ascertain the long axis of linear bones, such as the femur and pelvis. This analysis reveals untapped information on the spatial localization and organotropism trends exhibited by MM tumor cells.

Applying the proposed pipeline to a longitudinal preclinical PET/CT study demonstrated that osseous progression of MM in our model is influenced by the sex of the mouse. We observed a faster increase in PET signals (SUV) in female mice at earlier stages (day 18 post-tumor inoculation) and bone loss in the hip area at later time points (days 25 and 32). Tumor lesions exhibited a preference for certain areas aligned with joints. This granular data makes it feasible to investigate the progression of lesions in anatomical detail, offering significant insights into the role of the bone microenvironment and location-related weight-bearing patterns in MM, with an emphasis on the pelvis as a crucial contributor. It also provides valuable insights into tumor aggressiveness and local sex differences.

This study is limited by a small sample size, necessitating larger experiments for further validation. Motion artifacts around the femur area compromised the temporal analysis of femoral CT features. The projection analysis assumed stable bone shapes over time, which may not apply to all MM cases. Additionally, the application of PCA-generated linear projections is limited to linear bones. Curvier structures, such as the spine, lack meaningful biological interpretation with linear projections. We also excluded some bones, such as the skull, upper extremities, and cervical vertebrae. Future studies will be designed to overcome these limitations and will focus on extracting disease-specific radiomic features as well as developing prediction metrics for prognosis and treatment planning.

### Conclusion.

To quantify and compare multiple preclinical PET/CT scans, we developed a quantification pipeline that includes the following components: a deep learning based CT-guided bone segmentation model, an algorithm to mask potentially affected areas around the bladder and rectum to avoid false positives, and a projection-based aggregated analysis tool to temporally track PET/CT signal changes in myeloma-prone, bone marrow-rich skeletal sites. This pipeline allowed us to conveniently track SUV and HU signal changes over different regions of interest, including the pelvis, with detailed spatial information. In our proof-of-principle application, we observed a faster increase in PET signals (SUV) in all traced ROIs for female mice at earlier stages (day 18 post-tumor inoculation) and more bone loss in the hip area for females at later time points (days 25 and 32). Additionally, tumor lesions exhibited a preference for certain areas aligned with joints.

## Supplementary Files

This is a list of supplementary les associated with this preprint. Click to download.

- 20250508SupplementalFileEJNMMISun2025.pdf

## Figures and Tables

**Table 1.** Summary of the in-house dataset sizes.

Energy	Train	Valid	Test	Total
**80**	18	3	7	28
**60**	15	3	6	24
**80 + 60**	33	6	13	52

**Table 2.** Evaluation metrics for each ROI on the test data include Dice and mIoU, which assess the overlap between the segmented mask and the ground truth. Higher values indicate better performance, with a maximum score of 1 for all metrics.

Region	Dice	mIoU	Recall	Precision
Spine	0.963 ± 0.006	0.928 ± 0.011	0.957 ± 0.012	0.968 ± 0.012
R Femoral Body	0.974 ± 0.008	0.949 ± 0.016	0.964 ± 0.018	0.984 ± 0.014
L Femoral Body	0.977 ± 0.007	0.954 ± 0.013	0.970 ± 0.015	0.984 ± 0.010
PPJ	0.959 ± 0.012	0.921 ± 0.022	0.964 ± 0.015	0.954 ± 0.016
Sacrum	0.955 ± 0.006	0.914 ± 0.012	0.956 ± 0.016	0.955 ± 0.019
Spine + Sacrum	0.965 ± 0.006	0.932 ± 0.010	0.960 ± 0.011	0.970 ± 0.012
PPJ + Sacrum	0.962 ± 0.010	0.928 ± 0.018	0.966 ± 0.008	0.959 ± 0.014

**Table 3.** P-values from t-tests comparing changes in SUV metrics in the given ROI at day 18 with baseline scans between different sexes are presented. P-values less than 0.05 are highlighted in red, while others are shown in black. We observed that, for almost all metrics compared, female mice exhibit larger SUV changes, which may indicate more aggressive lesion development in the ROIs for females in the early stages. (Note: t-values are not shown here.)

Region	Mean	Medium	Quantile 75	Quantile 90	Quantile 95	Max
Spine	0.012	0.018	0.013	0.008	0.006	0.012
R Femoral Body	0.007	0.006	0.006	0.012	0.019	0.078
L Femoral Body	0.006	0.007	0.012	0.011	0.012	0.038
PPJ	0.018	0.009	0.018	0.039	0.067	0.121
Sacrum	0.02	0.026	0.019	0.019	0.025	0.068
Spine + Sacrum	0.012	0.016	0.013	0.007	0.005	0.005
PPJ + Sacrum	0.016	0.011	0.011	0.027	0.052	0.121

**Table 4.** The p-values from t-tests comparing changes in SUV and HU metrics in the acetabular segment of the pelvis between different sexes since baseline scans. P-values less than 0.05 are highlighted in red, while others are shown in black. In this context, “300<%<800” refers to the percentage of voxels with HU values between 300 and 800, representing trabecular bone, and “%>1000” indicates the percentage of cortical bone. The data suggest that female mice experience greater bone loss in the late stages of MM, as evidenced by p-values less than 0.05 for changes in HU_mean_ and HU_std_ between males and females at days 25 and 32.

Day	HU					SUV			
	300<%<800	%>1000	HU_max_	HU_mean_	HU_std_	%>2.5	SUV_max_	SUV_mean_	SUV_std_
11	0.884	0.283	0.073	0.101	0.488	0.356	0.139	0.159	0.136
18	0.277	0.053	0.687	0.069	0.010	0.227	0.012	0.014	0.025
25	0.007	0.006	0.101	0.008	0.002	0.431	0.188	0.215	0.191
32	0.297	0.064	0.250	0.048	0.033	0.279	0.136	0.149	0.115

## Data Availability

All data generated or analyzed during this study are included in the main manuscript and supplemental file. Once published, all data will be made publicly available via the publication.

## Key points.

### Question:

Can the proposed standardized PET/CT quantification pipeline add actionable biological insights to preclinical Multiple Myeloma imaging studies?

### Pertinent Findings:

To quantify and compare multiple preclinical PET/CT scans, we developed an AI-based quantification pipeline that includes a CT-guided bone segmentation model, an algorithm to mask potentially affected areas around the bladder and rectum to avoid false positives, and a projection-based aggregated analysis tool to localize PET/CT signal changes. In our proof-of-principle study, we observed a faster increase in PET signals (SUV) in all traced ROIs for female mice at earlier stages (day 18 post-tumor inoculation) and more bone loss in the hip area for female mice at later time points (days 25, 32). Additionally, tumor lesions exhibited a preference for certain areas aligned with joints.

### Implications for Patient Care:

Our proposed pipeline offers more granular data analysis, enabling the investigation of lesion progression within anatomical details, the role of the bone microenvironment, location-related weight-bearing patterns in MM, and local sex differences.

## Abbreviations.

MM: Multiple Myeloma
SUV: Standardized Uptake Value
HU: Hounsfield Units
IMWG: International Myeloma Working Group
ROI: Region of Interest
PCA: Principal Component Analysis
PPJ: Pelvis & Pelvic Joints
NIH: National Institutes of Health

### Supplementary Files

SPEP: Serum Protein Electrophoresis
radio-HPLC: radio-High-Performance Liquid Chromatography
IRW: Inveon Research Workplace
